# Aortic Elasticity and Cardiac Function in Fetuses With Aortic Coarctation

**DOI:** 10.3389/fcvm.2022.870683

**Published:** 2022-05-17

**Authors:** Dan Zhou, Ran Xu, Jiawei Zhou, Li Xie, Ganqiong Xu, Minghui Liu, Shi Zeng

**Affiliations:** ^1^Department of Ultrasound Diagnosis, The Second Xiangya Hospital, Central South University, Changsha, China; ^2^Department of Urology, The Second Xiangya Hospital, Central South University, Changsha, China; ^3^Department of Cardiology and Cardiovascular Surgery, The Second Xiangya Hospital, Central South University, Changsha, China

**Keywords:** aortic elasticity, aortic strain, aortic coarctation, cardiac function, echocardiography

## Abstract

**Objective:**

The purpose of the study was to observe the elasticity of the ascending aorta (AAo) in normal fetuses and fetuses with coarctation of the aorta (CoA) by M-mode echocardiography.

**Methods:**

This was a prospective clinical study performed on 16 fetuses with CoA and 48 gestational-age matched normal fetuses. The minimum internal diameter in the diastolic phase (Dmin) and the maximum internal diameter in the systolic phase (Dmax) of the AAo were measured by M-mode echocardiography. The aortic strain was calculated using the formula 100 × (*Dmax*−*Dmin*)/*Dmin*). Doppler echocardiography was performed to measure the cardiac function parameters. Correlations between aortic strain and cardiac function were assessed in fetuses with CoA.

**Results:**

The aortic strain of the ascending aorta in the fetuses with CoA was significantly lower than that in normal fetuses (18.12 ± 4.88% vs. 25.22 ± 4.92%, *p* < 0.01). The fetuses with CoA showed significantly higher combined cardiac output than the controls (471.89 ± 93.98 vs. 411.57 ± 46.35 ml/min/kg, *p* < 0.05). Compared with the normal group, the early diastolic velocities (E') and peak systolic velocities (S') of the left side were obviously decreased in the CoA group (*p* < 0.05), while the left early diastolic velocity ratio (E/E') was significantly increased in the fetuses with CoA (*p* < 0.01). For the fetuses with CoA, the aortic strain of the AAo was correlated with the left E/E' and S' (*r* = −0.522 and 0.504, respectively, *P* < 0.05).

**Conclusions:**

The aortic strain of the ascending aorta was significantly decreased in fetuses with CoA in middle-late gestation. The impaired strain of the ascending aorta was correlated with the left ventricle function in the fetuses with CoA. These findings imply that the abnormalities of the intrinsic aortic wall of CoA might develop early *in utero*.

## Introduction

Coarctation of the aorta (CoA) accounts for approximately 7% of all live births with congenital heart diseases and is defined as the narrowing of the aortic isthmus and the aortic arch to a variable degree (1). CoA is also the most common cause of hypertension among patients with congenital heart disease, and persistent hypertension still emerges in up to 50% of patients after CoA repairment (2). Abnormal aortic pulse propagation and altered elastic properties have been demonstrated by experimental animal models with coarctation of the aorta (3). Previous studies have demonstrated that children and adults with coarctation of the aorta, even after successful repair, have increased stiffness and decreased distensibility and compliance of the segment proximal to the coarctation (4–7). However, due to the special fetal hemodynamics and the inability of fetal blood pressure, the characteristics of aortic elasticity in fetuses with CoA has not been well elaborated. A study of the elastic properties of the ascending aorta in newborns with coarctation suggested that coarctation might be a systemic vascular disease of the precoarctational arteries (8). Whether these alterations in aortic elasticity in patients with coarctation develop early in fetal life is still unknown.

Interactions between the heart and the arterial system, the “ventricular–arterial coupling”, could be a crucial determinant of cardiovascular performance (9). Recent studies have presented variable results on the changes in the ventricular structure and function in fetuses with aortic coarctation (10, 11). Increased vascular stiffness has been shown to be associated with left ventricular diastolic function in adult and pediatric patients after the repair of aortic coarctation (4, 12). However, the interaction of these systems in fetuses with CoA *in utero* is not clear. The investigation of the aortic elasticity *in utero* may be beneficial to further explore the pathophysiology of aortic coarctation, which has not yet been completely elucidated.

The aortic strain has been used to assess the aortic elasticity in previous studies (13–15). The purpose of the study was to observe the aortic strain of the ascending aorta (AAo) in normal fetuses and fetuses with CoA by echocardiography and to investigate the correlation between arterial parameters and cardiac function.

## Methods

A prospective study was conducted in the Second Xiangya Hospital of Central South University in China between February 2021 and December 2021. Written informed consent was obtained from all families, and the study was approved by the Ethics Committees of the Second Xiangya Hospital.

Cases were recruited from fetuses with high echocardiographic suspicion of CoA who were evaluated in our department, including those with significant ventricular disproportion, an aortic isthmus diameter of z-score < −2, and reversed or mixed flow at the aortic arch (16). These cases postnatally confirmed the coarctation of the aorta. The exclusion criteria included the following: (1) multiple-gestation pregnancies; (2) small size for gestational age, defined as the estimated fetal weight (EFW) below the 10th percentile for fetuses of the same gestational age; (3) fetuses with the diagnosis of additional major cardiac malformation, extracardiac anomalies, and genetic anomalies; (4) identifiable chromosomal abnormalities or syndromes; (5) persistent fetal arrhythmia; and (6) maternal complications, including gestational diabetes, preeclampsia, and thyroid diseases. Gestation-matched normal pregnancies were collected as controls.

Routine obstetrical ultrasound and complete echocardiography were performed for each fetus by one operator using a Voluson E8 system (GE Healthcare, Milwaukee, WI, USA) with an RAB 4–8-D curvilinear probe. Gestational age (GA) was calculated based on the crown-rump length obtained at the first-trimester ultrasound. Fetal biometry was measured, and the EFW was calculated using Hadlock's formula (17). Standard and multiple views of each fetal heart were obtained to evaluate the cardiac anatomy. A pulsed Doppler examination of the umbilical artery (UA) was performed in a free loop of the umbilical cord. The pulsatility index of the middle cerebral artery (MCA) was measured at the proximal segment after its origin from the circle of Willis.

### Echocardiography of Aortic Strain

**Figure 1:**
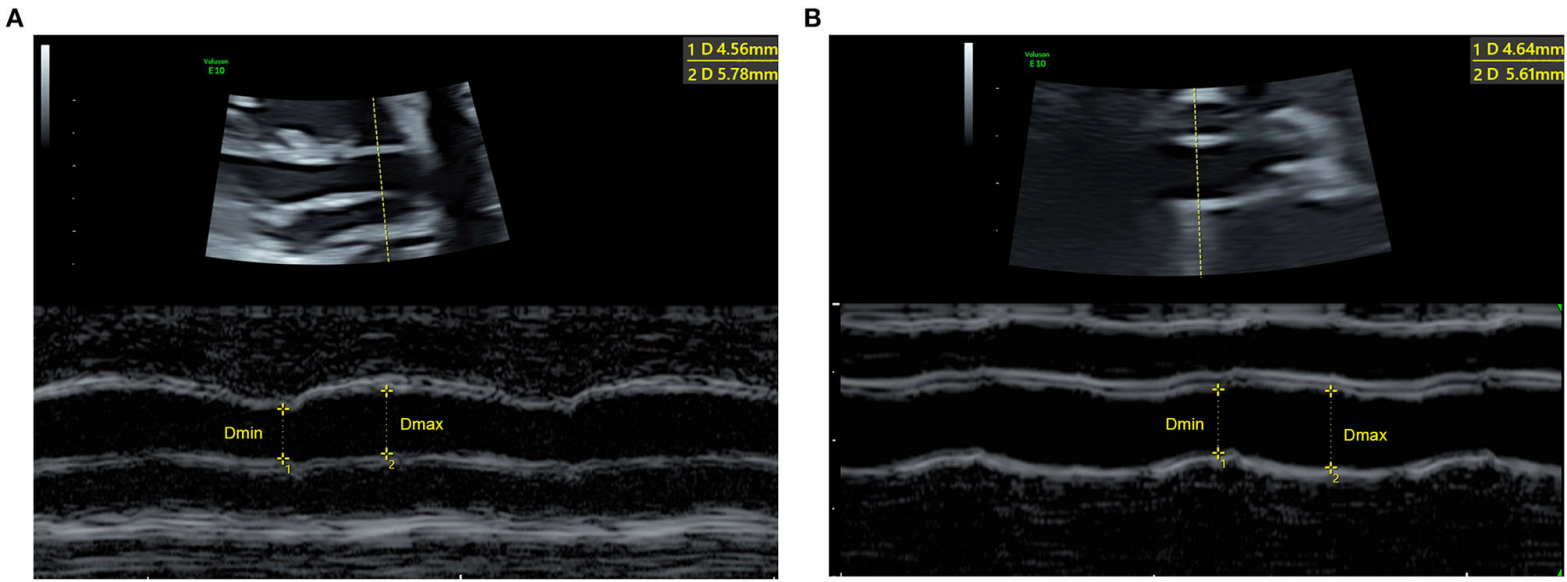
Measurement of the maximum and minimum internal aortic diameters to assess aortic strain from the M-mode echocardiography of a 32-week normal fetus **(A)** and a 33-week fetus with CoA **(B)**. Dmin, the minimum internal diameter in the diastolic phase, Dmax, the maximum internal diameter in the systolic phase.

The elasticity of the ascending aorta was assessed by M-mode echocardiography to evaluate vessel strain (Figure 1). The minimum internal diameter in the diastolic phase (Dmin) and the maximum internal diameter in the systolic phase (Dmax) were measured using M-mode echocardiography. The aortic strain was calculated as the formula: 100 × (*Dmax*−*Dmin*)/*Dmin*(13, 15). The fetal ascending aorta was measured below the innominate artery and above the sinotubular junction. The image was then enlarged as appropriate (with the AAo occupying at least 30% of the screen). The ultrasound sampling line was kept close to 90° to the aortic wall. All measurements were performed in resting fetuses, and fetal motion, such as breathing movements, was avoided. All echocardiographic measurements were averaged from 3 consecutive heartbeats.

### Echocardiography and Doppler Evaluations of Cardiac Function

Fetal cardiac morphometric measurements included diameters of atrio-ventricular valves and ventricular end-diastolic dimension of right and left ventricle. Cardiac dimensions were measured at their maximal size from inner-edge to inner-edge. At least three estimates of each dimension were taken from separate frames and the mean was used. Systolic function evaluation included the heart rate (HR), right and left stroke volumes (SV), cardiac output (CO), and peak systolic myocardial velocities (S'). Left and right SV were calculated as π/4 × *d*^2^×*TVI*, where d is the diameter of the aortic or pulmonary artery valve (18). The aortic or pulmonary artery velocity-time integrals were calculated by manual tracing of the spectral Doppler area of the LV outflow tract wave. Cardiac output was calculated as SV^*^HR and was further adjusted by the estimated fetal weight (18, 19). Diastolic function was assessed by ventricular inflow peak velocities in early diastole (E) and atrial contraction (A), the E/A ratio, the peak myocardial velocity in early diastole (E'), and the early diastolic velocity ratio (E/E'). Atrioventricular flows were obtained from a basal or apical 4-chamber view, with the pulsed Doppler sample volume placed just below the valve leaflets. The insonation angle was kept below 10°. The sample volume was set at 1 to 3 mm; the high-pass filter was set at 100 to 200 Hz. The E and A velocities were measured and the E/A ratio was calculated as a percentage of the mean duration of 3 cardiac cycles. For tissue Doppler imaging, sample volumes were placed in the basal part of the mitral annulus and tricuspid annulus in a 4-chamber view. The ultrasound beam was kept at an angle of <30° to the orientation of the ventricular wall or interventricular septum. No angle correction was applied. The E', S' were measured, and the E/E' ratio was calculated (20). All Doppler evaluations were carried out in the absence of fetal breathing and body movements. Each measurement was repeated three times, and the mean was used for further analyses.

### Statistics

All the data are reported as means with standard deviations or frequencies with percentages, as appropriate. The Shapiro-Wilk test of normality was performed for the continuous variables. Comparisons of the clinical features and echocardiographic parameters were performed between the fetuses with CoA and the controls using Student's *t*-test or the Mann-Whitney U test or the Chi-square analysis. Correlations between the aortic strain and cardiac function parameters were tested using Pearson or Spearman correlation coefficients. To assess interobserver variability, the aortic strains were independently measured by a second reader who was blinded to the clinical data of 20 normal fetuses and 10 fetuses with CoA that were randomly selected. To assess intraobserver variability, a single observer then analyzed the data arising from these cases two times, with one-day intervals between the analyses. A probability value of *P* < .05 was considered to indicate a statistically significant difference. All statistical analyses were performed using PASW statistical software [PASW (SPSS) statistics, version 20.0, IBM] and GraphPad Prism 8.

## Results

### General Condition

In all, 75 fetuses were enrolled during the study period. Six and 4 fetuses were excluded due to excessive fetal motion and loss of follow-up, respectively, and one fetus suspected to have CoA was ruled out due to a normal postnatal echocardiography. A total of 64 fetuses were finally included: 16 fetuses with aorta coarctation and 48 gestational age-matched normal fetuses. Table 1 lists the clinical demographics and aortic parameters of all the enrolled fetuses. The gestational age at scan for the fetuses with CoA and those in the normal control group were 30.0 ± 3.7 and 30.2 ± 3.4 weeks, respectively. There was no significant difference in the UA-PI and MCA-PI between the two groups. The GA at birth and birth weight of the CoA group were smaller than those of the control group. Thirteen cases underwent cardiac surgery at a mean age of 19 ± 5 days. Two infants died during the peri-surgical period (within 30 days of surgery).

**Table 1:** Clinical data and Echocardiography of artery and cardiac function in the controls and fetuses with CoA.

**Variable**	**Controls (*n* = 48)**	**Fetus with CoA (*n* = 16)**	**P-value**
Maternal age (years)	28.8 ± 2.6	28.4 ± 4.1	0.944
AR pregnancy	7 (15%)	4 (25%)	0.566
GA at diagnosis (weeks)	30.0 ± 3.7	30.2 ± 3.4	0.889
EFW at diagnosis (g)	1590 ± 508	1460 ± 489	0.288
MCA-PI	1.81 ± 0.25	1.80 ± 0.23	0.901
UA-PI	0.88 ± 0.14	0.87 ± 0.11	0.889
**M-mode echocardiography of artery**			
**Ascending aorta**			
Dmax (mm)	5.67 ± 0.85	5.00 ± 1.01	0.011
Dmin (mm)	4.54 ± 0.70	4.22 ± 0.75	0.128
Aortic Strain (%)	25.22 ± 4.92	18.12 ± 4.88	<0.001
**Echocardiography and Doppler of cardiac function**			
Combined cardiac output (ml/min/kg)	411.57 ± 46.35	471.89 ± 93.98	0.014
HR (bpm)	143.6 ± 8.2	144.8 ± 10.2	0.657
**Left ventricle dimensions**			
Mitral annulus diameter (mm)	9.37 ± 1.57	7.72 ± 1.39	<0.001
Mitral annulus diameter (z-score)	−0.11 ± 0.44	−1.59 ± 0.77	<0.001
LV end-diastolic diameter (mm)	10.46 ± 1.96	8.72 ± 1.77	0.002
LV end-diastolic diameter (z-score)	−0.44 ± 0.39	−2.08 ± 0.73	<0.001
**Right ventricle dimensions**			
Tricuspid annulus diameter (mm)	10.29 ± 1.89	10.11 ± 2.21	0.754
Tricuspid annulus diameter (z-score)	0.04 ± 0.42	−0.10 ± 0.77	0.467
RV end-diastolic diameter (mm)	11.84 ± 1.96	12.02 ± 2.44	0.78
RV end-diastolic diameter (z-score)	−0.19 ± 0.43	−0.39 ± 0.81	0.363
**Left ventricle functional parameters**			
SV (ml)	2.25 ± 0.71	1.38 ± 0.64	<0.001
CO (ml/min)	320.65 ± 94.90	198.75 ± 88.21	<0.001
Cardiac output by EFW (ml/min/kg)	207.70 ± 36.47	135.06 ± 37.01	<0.001
E (cm/s)	36.23 ± 6.33	39.34 ± 6.43	0.094
A (cm/s)	56.19 ± 8.21	59.80 ± 8.21	0.133
E/A	0.65 ± 0.07	0.65 ± 0.08	0.853
E' (cm/s)	5.71 ± 1.27	4.93 ± 1.12	0.034
E/E'	6.55 ± 1.42	8.32 ± 2.17	<0.001
S' (cm/s)	5.63 ± 1.19	4.54 ± 1.11	0.002
**Right ventricle functional parameters**			
SV (ml)	2.25 ± 0.70	3.38 ± 1.57	0.002
CO (ml/min)	320.42 ± 97.61	507.67 ± 247.57	0.002
Cardiac output by EFW (ml/min/kg)	203.87 ± 26.23	340.84 ± 98.37	<0.001
E (cm/s)	41.90 ± 6.74	46.69 ± 8.69	0.026
A (cm/s)	56.66 ± 8.73	63.51 ± 12.07	0.055
E/A	0.74 ± 0.07	0.74 ± 0.08	0.856
E' (cm/s)	6.87 ± 0.86	6.74 ± 1.66	0.75
E/E'	6.13 ± 0.82	7.38 ± 2.43	0.07
S' (cm/s)	5.9 ± 0.83	5.99 ± 1.07	0.732
**Postnatal outcome**			
GA at birth (weeks)	39.0 ± 0.89	37.9 ± 0.96	<0.001
Birth weight (g)	3214 ± 289	2884 ± 274	0.001
Admission to NICU	0 (0%)	12 (75%)	-
Before-surgery death, n (%)	-	0 (0%)	-
Peri-surgery death, *n* (%)	-	2 (12.5%)	-
cardiac surgery, *n* (%)	-	13 (81.2%)	-
cardiac surgery at age (days)	-	18.8 ± 5.4	-
Length of hospital stay (days)	-	30.9 ± 4.4	-

*CoA, coarctation of the aorta; AR, assisted reproduction; GA, gestation age; EFW, estimated fetal weight; UA-PI, umbilical artery pulsatility index; MCA-PI, middle cerebral artery pulsatility index; NICU, neonatal intensive care unit; SV, stroke volumes; CO, cardiac output; LV, left ventricle; RV, right ventricle*.

### Aortic Strain and Cardiac Function Parameters

**Figure 2:**
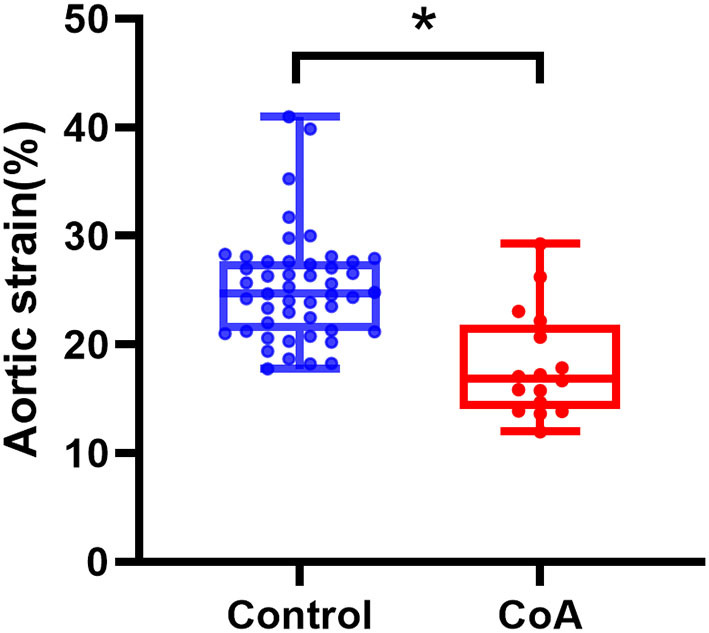
Aortic strain of ascending aorta in the controls and the fetuses with CoA. Compared with the normal group, *p < 0.05. CoA, coarctation of the aorta.

The Dmax of the AAo in the fetuses with CoA was significantly lower than that in controls (5.67 ± 0.85 vs. 5.00 ± 1.01 mm, *p* < 0.05). The aortic strain of the ascending aorta in the fetuses with CoA was significantly lower than that in normal fetuses (18.12 ± 4.88% vs. 25.22 ± 4.92%, *p* < 0.01). However, there was no significant difference found in the Dmin of the AAo between the two groups (Table 1 and Figure 2).

Compared with the controls, the CoA group exhibit significant lower values of the diameters of both mitral annulus and left ventricle (*p* < 0.01). The left-side cardiac output in the group with CoA was significantly lower than that of the control group (135.06 ± 37.01 vs. 207.70 ± 36.47 ml/min/kg, *p* < 0.01). The CO of the right side in the fetuses with CoA was significantly increased compared with that of fetuses in the control group (340.84 ± 98.37 vs. 203.87 ± 26.23 ml/min/kg, *p* < 0.01). The fetuses with CoA showed significantly higher combined cardiac output than controls (471.89 ± 93.98 vs. 411.57 ± 46.35 ml/min/kg, *p* < 0.05). Compared with the normal group, the early diastole velocities (E'), and peak systolic velocities (S') of the left side were decreased in the CoA group (*p* < 0.05), while the E/E' was significantly increased in the fetuses with CoA (*p* < 0.01). No difference was shown in the right ventricle function parameters between the two groups (Table 1).

**Figure 3:**
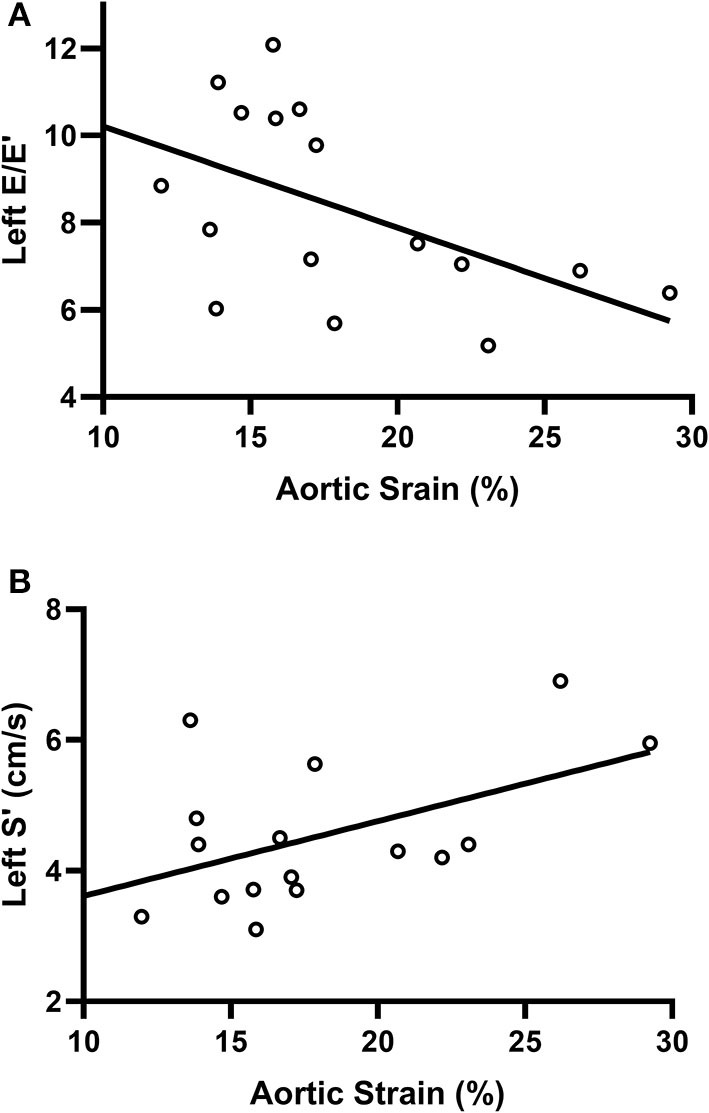
Regression curves demonstrating the association of aortic strain with left E/E'**(A)** and S'**(B)** in the fetuses with CoA. There was a negative correlation between the strain of the AAo and left E/E' and a positive correlation between aortic strain and left S' in the CoA group (*r* = −0.522 and 0.504, respectively, *P* < 0.05). S', peak systolic myocardial velocities, E/E', the early diastolic velocity ratio.

The correlation between the aortic strain and cardiac function was shown in Figure 3. For the fetuses with CoA, the strain of the AAo was correlated with the left E/E' and S' (*r* = −0.522 and .504, respectively, *P* < 0.05), and no association was shown between aortic strain and cardiac function of the right side.

### Reproducibility of Aortic Strain

The intraclass correlation coefficients of interobserver and intraobserver for the aortic strain of AAo were .872 (95% CI, .75 −0.937) and .893 (95% CI, .789–0.947), respectively.

## Discussion

This study found that the aortic strain of the AAo in the fetuses with CoA was significantly lower than that in normal fetuses. For fetuses with CoA, the aortic strain of AAo was negatively correlated with the left E/E' and positively correlated with the left S'. To the best of our knowledge, this study is the first to demonstrate impaired aortic elasticity and its relation with cardiac function parameters in fetuses with CoA.

In this study, the aortic strain of the ascending aorta in fetuses with CoA was lower than that in normal fetuses, suggesting a decreased ascending aorta elasticity of fetuses with CoA in middle-late gestation. In fact, elastic properties of the aorta mostly depend on the presence of scleroprotein in the vessel wall, including elastin and collagen (21). Through *in vitro* experiments of the human aortas in patients aged 6–35 years, Sehested et al. found significantly more collagen and less smooth muscle mass in the proximal segment and within the coarctation than in the distal segment (22). We might suppose that the impaired strain of the ascending aorta in the CoA group might be related to the distribution and the accumulation of elastin and collagen. The impaired elasticity of the AAo in our study was consistent with previous studies of aortic elasticity in neonates and children with preoperative coarctation. Xu et al. applied intracavitary ultrasound in children with aortic coarctation and found significantly increased stiffness and decreased distensibility and compliance of the segment proximal to the coarctation compared with the segment of the aorta distal to the coarctation (23). Vogt et al. presented that the aortic elastic properties of the ascending aorta are primarily impaired in newborns with coarctation preoperatively (8). Our study implied that the intrinsic aortic wall abnormalities of fetuses with CoA might develop early *in utero*.

In this study, we found that the AS of the ascending aorta was negatively correlated with left E/E' and positively correlated with left S' in fetuses with CoA. Compared with the control group, a significantly higher E/E' and lower E' of the mitral annulus were found in the CoA group, which implied the higher filling pressure of the left ventricle. The decreased strain of the ascending aorta was related to the higher filling pressures of the left ventricle. Lombardi et al. found that the relation between proximal aortic stiffness and diastolic function was stronger than that for the corresponding distal segment in children after CoA repair (12). Our results showed a similar association between aortic strain and left ventricular diastolic function. Impaired vascular elasticity indirectly leads to impaired early diastolic relaxation (12), which corresponds to the higher filling pressures. Our finding suggest that the effect of decreased aortic elasticity on diastolic function occurs earlier in fetal life. Our results showed that the left side S' was decreased in fetuses with CoA, suggesting increased afterload. The positive correlation between the strain of the AAo and S' is homologous to the relation between the impaired aortic elasticity and increased afterload. Our results demonstrating that the positive correlation between decreased aortic elasticity and afterload in fetuses with CoA was similar to that after birth. A previous study showed that persisting hypertension was associated with the impaired elastic capacity of the aorta (24). Persistent hypertension is common in patients with CoA, both preoperatively and postoperatively. Ghorbani et al. found that, in young patients with coarctation, distensibility impairments of the aorta were predominantly associated with arterial hypertension (25).

This study has some limitations. First, this study only enrolled a small number of fetuses with CoA (*n* = 16) in middle-late gestation. Second, although the aortic strain obtained by M-mode echocardiography has been used to assess aortic elasticity in previous studies of children and adults (13, 14), when performed in fetuses, the results might be affected by small gestational age and fetal position. To minimize the impact, the depth, sample frame, and waveform speed were adjusted when obtaining the image. Third, as this was a cross-sectional study, all conclusions were based on a single middle-late trimester observation. More longitudinal studies are needed to further investigate these alterations.

In conclusion, the aortic strain of the ascending aorta was significantly decreased in fetuses with CoA compared with controls in middle-late gestation. The impaired strain of the ascending aorta was correlated with left ventricle function in fetuses with CoA. These findings might provide evidence for the assumption that coarctation is a systemic vascular disease of the prestenotic arteries and imply that the abnormalities of the intrinsic aortic wall of CoA might develop early *in utero*.

## Data Availability Statement

The raw data supporting the conclusions of this article will be made available by the authors, without unduereservation.

## Ethics Statement

The studies involving human participants were reviewed and approved by The Ethics Committees of the Second Xiangya Hospital. The patients/participants provided their written informed consent to participate in this study. Written informed consent was obtained from the individual(s) for the publication of any potentially identifiable images or data included in this article.

## Author Contributions

RX, LX, GX, and ML performed the ultrasonic image and collected the data. DZ and SZ analyzed the data and wrote the manuscript. All authors reviewed the final manuscript.

## Funding

This study was supported by the National Natural Sciences Foundation of China (nos. 81871372 and 81501497) and the Natural Science Foundation of Hunan Province (no. 2019JJ40425). The funding body was not involved in the design of the study and the collection, analysis, and interpretation of data and in the writing of the manuscript.

## Conflict of Interest

The authors declare that the research was conducted in the absence of any commercial or financial relationships that could be construed as a potential conflict of interest.

## Publisher's Note

All claims expressed in this article are solely those of the authors and do not necessarily represent those of their affiliated organizations, or those of the publisher, the editors and the reviewers. Any product that may be evaluated in this article, or claim that may be made by its manufacturer, is not guaranteed or endorsed by the publisher.
